# ZEB1 Upregulates VEGF Expression and Stimulates Angiogenesis in Breast Cancer

**DOI:** 10.1371/journal.pone.0148774

**Published:** 2016-02-16

**Authors:** Lingjia Liu, Qi Tong, Shuo Liu, Jianlin Cui, Quansheng Zhang, Wei Sun, Shuang Yang

**Affiliations:** 1 Tianjin Key Laboratory of Tumor Microenvironment and Neurovascular Regulation, Medical College of Nankai University, Tianjin 300071, China; 2 Tianjin Key Laboratory of Organ Transplantation, Tianjin First Center Hospital, Tianjin 300192, China; Medical College of Wisconsin, UNITED STATES

## Abstract

Although zinc finger E-box binding homeobox 1 (ZEB1) has been identified as a key factor in the regulation of breast cancer differentiation and metastasis, its potential role in modulating tumor angiogenesis has not been fully examined. Here, we present the novel finding that conditioned medium derived from ZEB1-expressing MDA-MB-231 cells significantly increased the capillary tube formation of human umbilical vein endothelial cells (HUVECs), whereas ZEB1 knockdown by RNA interference had the opposite effect. ZEB1 caused marked upregulation of the expression of vascular endothelial growth factor A (VEGFA) at both mRNA and protein levels. Pre-incubation of HUVECs with anti-VEGFA neutralized antibody attenuated ZEB1-mediated tube formation of HUVECs. In breast cancer tissues, expression of ZEB1 was positively correlated with those of VEGFA and CD31. At the molecular level, ZEB1 activated VEGFA transcription by increasing SP1 recruitment to its promoter, which was mediated via the activation of PI3K and p38 pathways. Using a nude mouse xenograft model, we demonstrated that elevated expression of ZEB1 promotes *in vivo* tumorigenesis and angiogenesis in breast cancer. Collectively, we found that ZEB1-expressing breast cancer cells increase VEGFA production and thus stimulate tumor growth and angiogenesis via a paracrine mechanism.

## Introduction

Tumor angiogenesis is characterized by the formation of new, irregular blood vessels from a preexisting vascular network. This abnormal angiogenesis is required for growth, survival, and metastasis of most solid tumors, involving the orchestrated action of many bioactive molecules [1,2]. The vascular endothelial growth factor (VEGF) family, which consists of 5 glycoproteins, namely vascular endothelial growth factor A (VEGFA), VEGFB, VEGFC, VEGFA-D (also known as c-fos-induced growth factors), and placental growth factor, is the best-characterized modulators of angiogenesis and metastatic growth in human carcinogenesis [3–5]. VEGF in breast cancer is associated with poor prognosis, as evidenced by the increased synthesis of this growth factor in breast cancer cells [6,7] as well as in breast cancer tissues [8]. VEGF stimulates proliferation and migration of naturally quiescent endothelial cells during tumor angiogenesis and growth, resulting in the formation of new vessel structures. Although hypoxia appears to be the main driver of tumor angiogenesis, growth factors and a variety of transcriptional regulators are able to boost VEGF production through paracrine or autocrine mechanisms [9–11]. However, much remains to be understood about the molecular nature of other factors that may also be able to promote angiogenesis in the development of breast cancer.

One such factor is zinc finger E-box binding homeobox 1 (ZEB1), a member of the zinc finger-homeodomain transcription factor family that modulates cell differentiation and tissue-specific cellular functions [12–15]. ZEB1 expression is implicated in the differentiation of multiple cell lineages, including bone- [16,17], smooth muscle [18,19], neural- [20], and T-cells [18,21,22]. Recent studies demonstrate that ZEB1 is a central regulator of the epithelial to mesenchymal transition (EMT) in solid tumors through transcriptional repression of E-cadherin [23–28]. In addition to repressing epithelial polarity and adhesion genes, ZEB1 activates mesenchymal and stemness markers [29,30]. Consequently, ZEB1 expression promotes tumorigenesis and metastasis in mouse models and correlates with a poorer prognosis in human cancers, including breast carcinomas. ZEB1 was recently implicated in pathological angiogenesis in lung cancer [31], although its exact mechanism of action is yet to be determined.

In the current study, we found that overexpression of ZEB1 is able to increase VEGFA synthesis in MDA-MB-231 cells and thus induces *in vivo* tumorigenesis and angiogenesis, characterized by increased formation of new blood vessels. Importantly, ZEB1 expression is positively correlated with VEGFA expression and blood vessel density in human breast cancer specimens. These data collectively provide evidence that ZEB1 plays a key role in regulating not only tumor growth but also pathologic angiogenesis in breast cancer.

## Results

### Ectopic expression of ZEB1 promotes tumor angiogenesis in breast cancer

**Fig 1:**
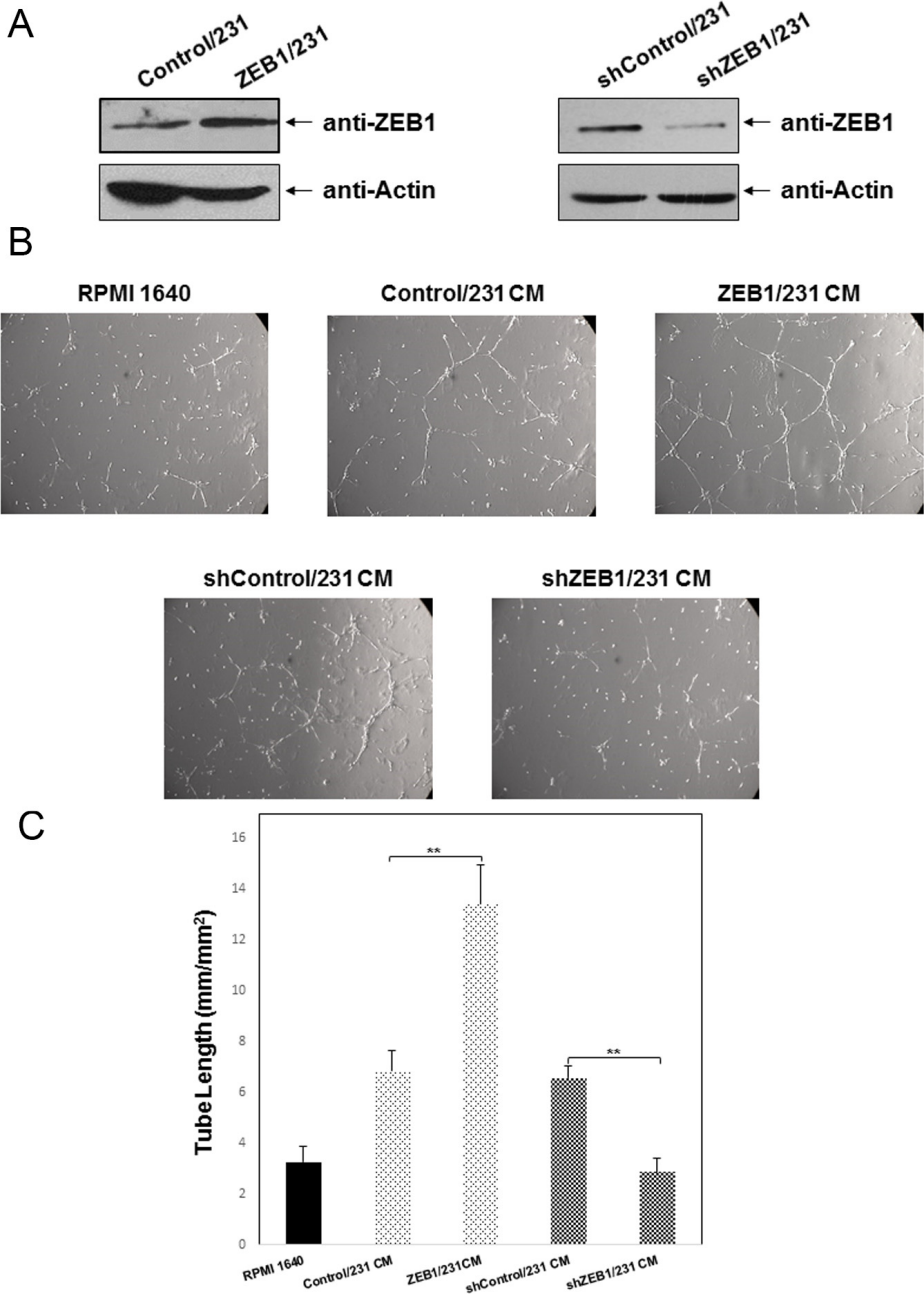
Ectopic expression of ZEB1 promotes MDA-MB-231-mediated angiogenesis *in vitro*. (A) MDA-MB-231 cells were stably transfected with the human ZEB1 expression plasmid (ZEB1/231) or shRNA plasmid targeting ZEB1 (shZEB1/231). Expression of ZEB1 protein was determined by Western blotting. Actin was used to normalize ZEB1 levels. (B) HUVECs cultured in ZEB1/231- or shZEB1/231-derived conditioned medium were subjected to a tube formation assay and photographed. (C) Quantification of tube formation was expressed as length of capillary tubes formed per mm^2^. **P < 0.01 vs respective control in Student’s *t*-test.

To assess the possible role of ZEB1 in breast cancer angiogenesis, MDA-MB-231 cells were stably transfected with either the full-length ZEB1 expression plasmid (ZEB1/231) or shRNA plasmid targeting ZEB1 (shZEB1/231). MDA-MB-231 cells stably transfected with empty vector (Control/231) or scramble shRNA (shControl/231) were used as controls. Overexpression or knockdown of ZEB1 was assessed by Western blotting (Fig 1A). HUVECs grown on Matrigel were then cultured in the conditioned medium derived from ZEB1/231 or shZEB1/231 cells, and their ability to induce tube formation was examined. As shown in Fig 1B, the capillary tube formation of HUVECs cultured in ZEB1/231-derived conditioned medium was significantly increased compared with those cultured in Control/231-derived medium. HUVECs grown in ZEB1/231-derived medium formed approximately 2-fold more capillary tubes than those grown in Control/231-derived medium (Fig 1C). ZEB1 depletion resulted in an opposite effect, inhibiting the capillary tube formation of HUVECs (Fig 1B and 1C) and highlighting that ectopic expression of ZEB1 may contribute to tumor angiogenesis in breast cancer.

### ZEB1 induces VEGFs expression

**Fig 2:**
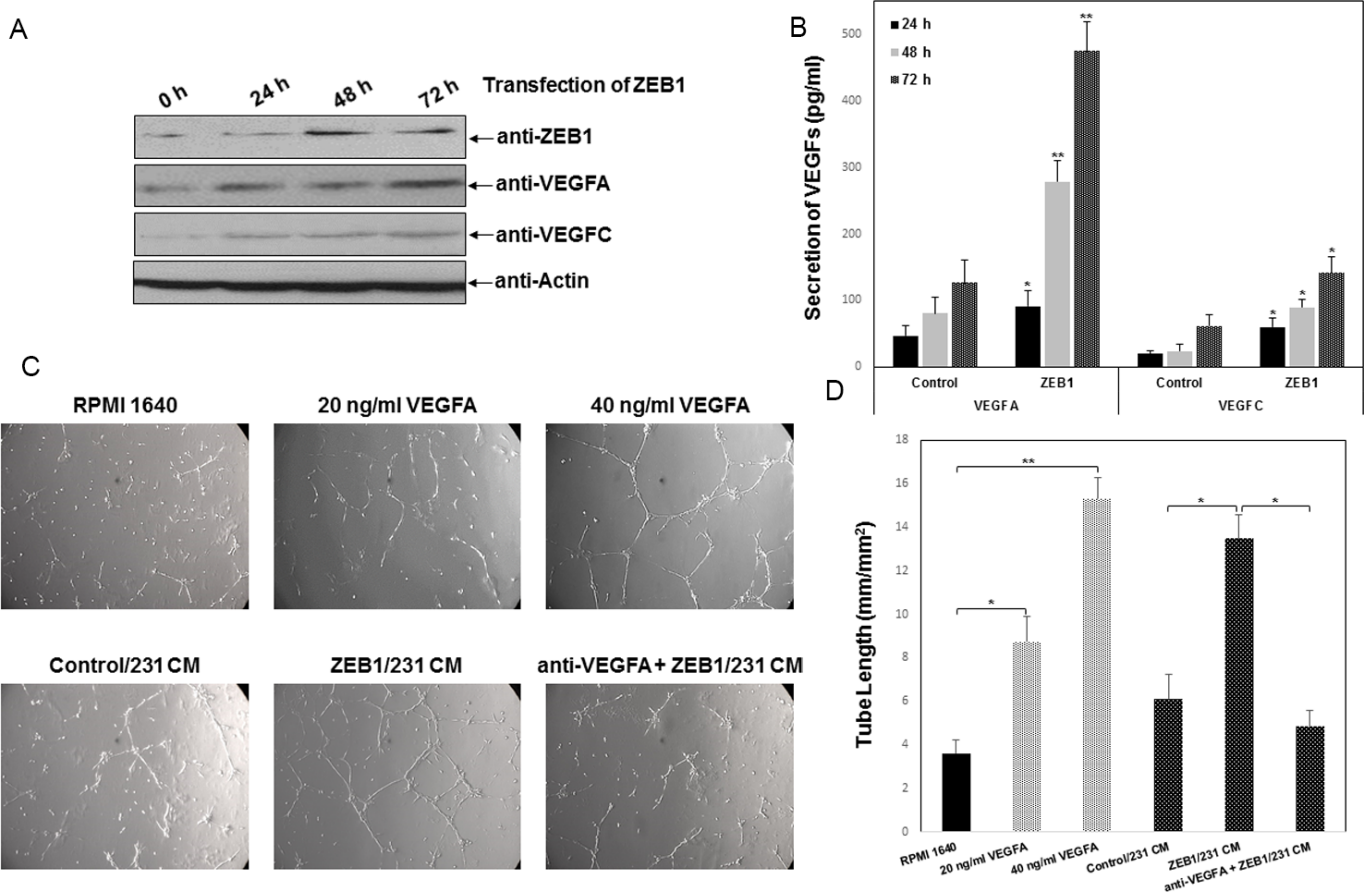
ZEB1 increases VEGFs secretion to induce tumor angiogenesis *in vitro*. (A) MDA-MB-231 cells were transiently transfected with human ZEB1 expression plasmid or empty vector control. At the indicated time points, expression of ZEB1, VEGFA and VEGFC was verified by Western blotting. Actin was used as a loading control. (B) Production of VEGFA and VEGFC protein were verified by ELISA at the indicated time points following ZEB1 overexpression. *P < 0.05, **P < 0.01 vs respective control in one-way ANOVA followed by Tukey’s Honestly Significant Difference test. (C) HUVECs cultured in the presence or absence of VEGFA (20 and 40 ng/mL) or anti-VEGFA neutralized Ab (1 μg/mL) along with ZEB1/231-derived conditioned medium were subjected to a tube formation assay and photographed. (D) Quantification of tube formation was expressed as length of capillary tubes formed per mm^2^. *P < 0.05, **P < 0.01 vs respective control in Student’s *t*-test.

To further explore the molecular changes, differences in the mRNA levels of selected molecules in angiogenesis signaling were examined using the RT^2^ Profiler PCR Array by comparing ZEB1-expressing cells with controls. Alterations in expression of several regulators, including EFNB2, VEGFA, VEGFC, PDGFA, and IL6, were observed (Table 1 and S1 Fig). Given that the VEGF family members are potent modulators of angiogenesis and metastatic growth in the pathophysiology of breast cancer, we further confirm the regulation of ZEB1 on VEGFs secretion by Western blotting and ELISA. The ZEB1 expression plasmid was transiently transfected into MDA-MB-231 cells. A significant, time-dependent increase of VEGFA production was observed following ZEB1 overexpression (Fig 2A and 2B). However, the alternation of VEGFC secretion was not as evident, implying a predominant role of ZEB1 to induce VEGFA expression. Thus, HUVECs grown on Matrigel were cultured in the presence or absence of VEGFA. As shown in Fig 2C and 2D, the ability of HUVECs to form capillary tubes in Matrigel was significantly increased by VEGFA addition. HUVECs grown in 20 and 40 ng/mL VEGFA formed approximately 2.4- to 4.2-fold more capillary tubes, respectively, than those grown in RPMI control medium. To demonstrate that ZEB1-induced expression of VEGFA contributes, at least partially, to tumor angiogenesis in breast cancer, HUVECs were pre-incubated with human anti-VEGFA-neutralized antibody (Ab) for 30 min and then cultured in ZEB1/231-derived conditioned medium. The capillary tube formation of HUVECs was dramatically reduced by 64% after the addition of anti-VEGFA Ab to ZEB1/231-derived conditioned medium (Fig 2C and 2D). Taken together, these observations suggest that ZEB1-expressing breast cancer cells have elevated VEGFA production and thus stimulate tumor angiogenesis via a paracrine mechanism.

**Table 1:** Genes regulated by ZEB1.

Unigene	GeneBank^TM^ accession no.	Symbol	Description	Fold change (ZEB1 vs. Control)	*P* value
Hs.149239	NM_004093	EFNB2	Ephrin-B2	5.5842	0.0218
Hs.73793	NM_003376	VEGFA	Vascular endothelial growth factor A	4.9148	0.0452
Hs.435215	NM_005429	VEGFC	Vascular endothelial growth factor C	4.0042	0.0173
Hs.535898	NM_002607	PDGFA	Platelet-derived growth factor alpha polypeptide	2.7401	0.0458
Hs.654458	NM_000600	IL6	Interleukin 6	2.2739	0.0351

### ZEB1 is associated with VEGFA expression and blood vessel density in human breast cancer

**Fig 3:**
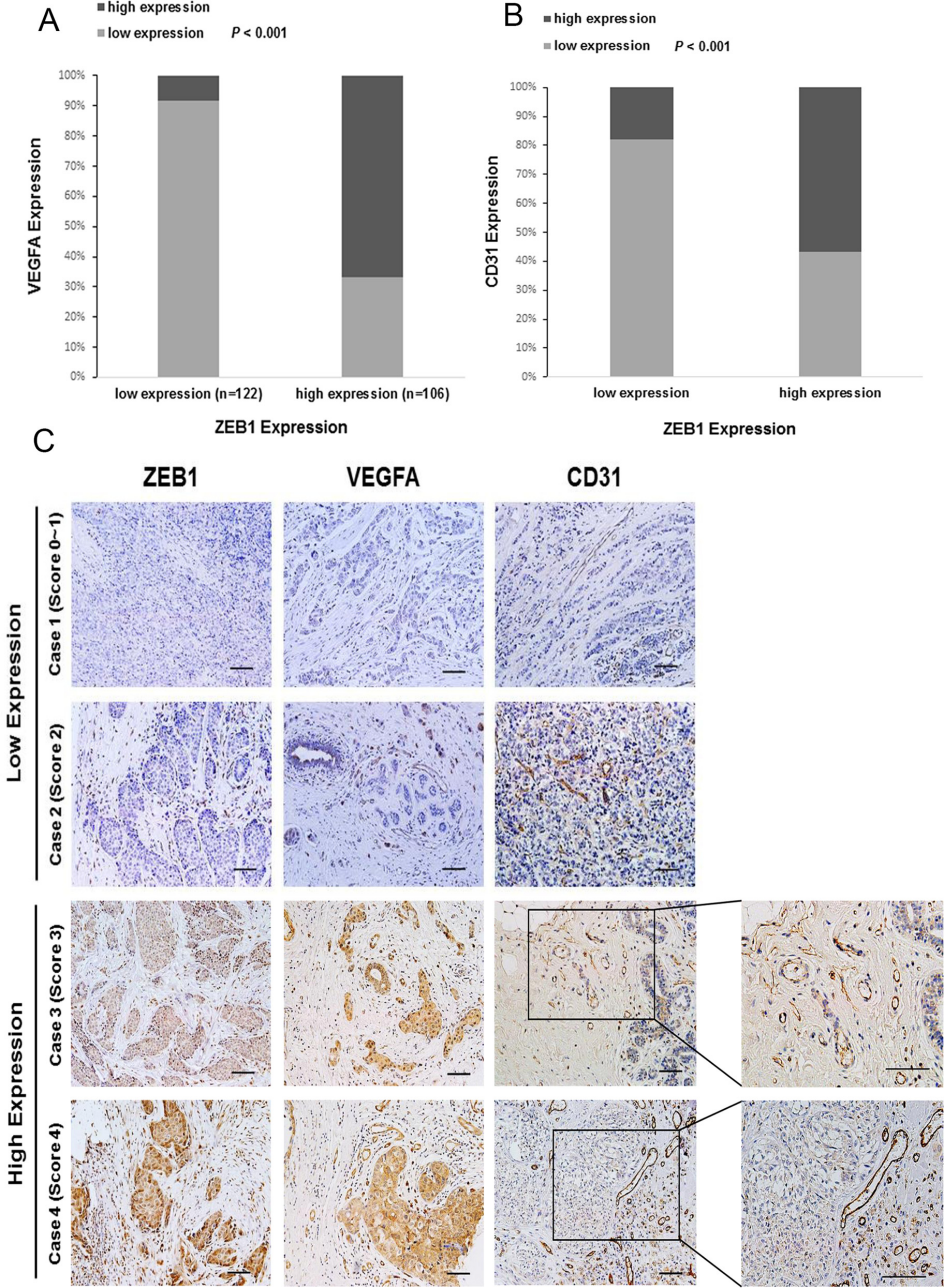
ZEB1 is associated with VEGFA expression and blood vessel density in human breast cancer. The non-negative percentage analysis for VEGFA (A) and CD31 (B) indicates a positive correlation with ZEB1 expression in breast cancer tumors from 228 subjects. (C) Representative images of immunohistochemical staining of ZEB1, VEGFA, and CD31 in tumors from 4 cases are shown. Scale bars, 25 μm.

To further substantiate a link between ZEB1, VEGFA and tumor angiogenesis, we performed immunohistochemical staining for ZEB1, VEGFA, and CD31 in 228 cases of human breast carcinoma. We found that cancers displaying a high percentage of ZEB1-positive cells exhibited a high level of VEGFA expression (Table 2 and Fig 3A) and CD31 density (Table 3 and Fig 3B). In contrast, cancers exhibiting little ZEB1 showed diminished VEGFA expression and vascularization. Pearson Chi-square analysis demonstrated that ZEB1 expression was positively correlated with VEGFA expression (*P* < 0.001; r = 0.613) and blood vessel density (*P* < 0.001; r = 0.401). Moreover, immunohistochemical staining of samples from 4 representative subjects confirmed the positive relationship between ZEB1, VEGFA, and CD31 expression (Fig 3C), which is consistent with our finding that ZEB1 upregulates VEGFA expression and promotes tumor angiogenesis in breast cancer.

**Table 2:** Positive correlation between ZEB1 and VEGFA expression in breast cancer.

VEGFA expression level	No. of IDC-NOS specimens	
	Low ZEB1 expression	High ZEB1 expression
Low VEGFA expression	112	35
High VEGFA expression	10	71
Total	122	106

*P* < 0.001; r = 0.613

*P* values were calculated by Spearman’s Rank-Correlation test (n = 228)

**Table 3:** Positive correlation between ZEB1 and CD31 expression in breast cancer.

CD31 expression level	No. of IDC-NOS specimens	
	Low ZEB1 expression	High ZEB1 expression
Low CD31 expression	100	46
High CD31 expression	22	60
Total	122	106

*P* < 0.001; r = 0.401

*P* values were calculated by Spearman’s Rank-Correlation test (n = 228)

### ZEB1 upregulates VEGFA expression by differentially regulating the PI3K and p38 pathways

**Fig 4:**
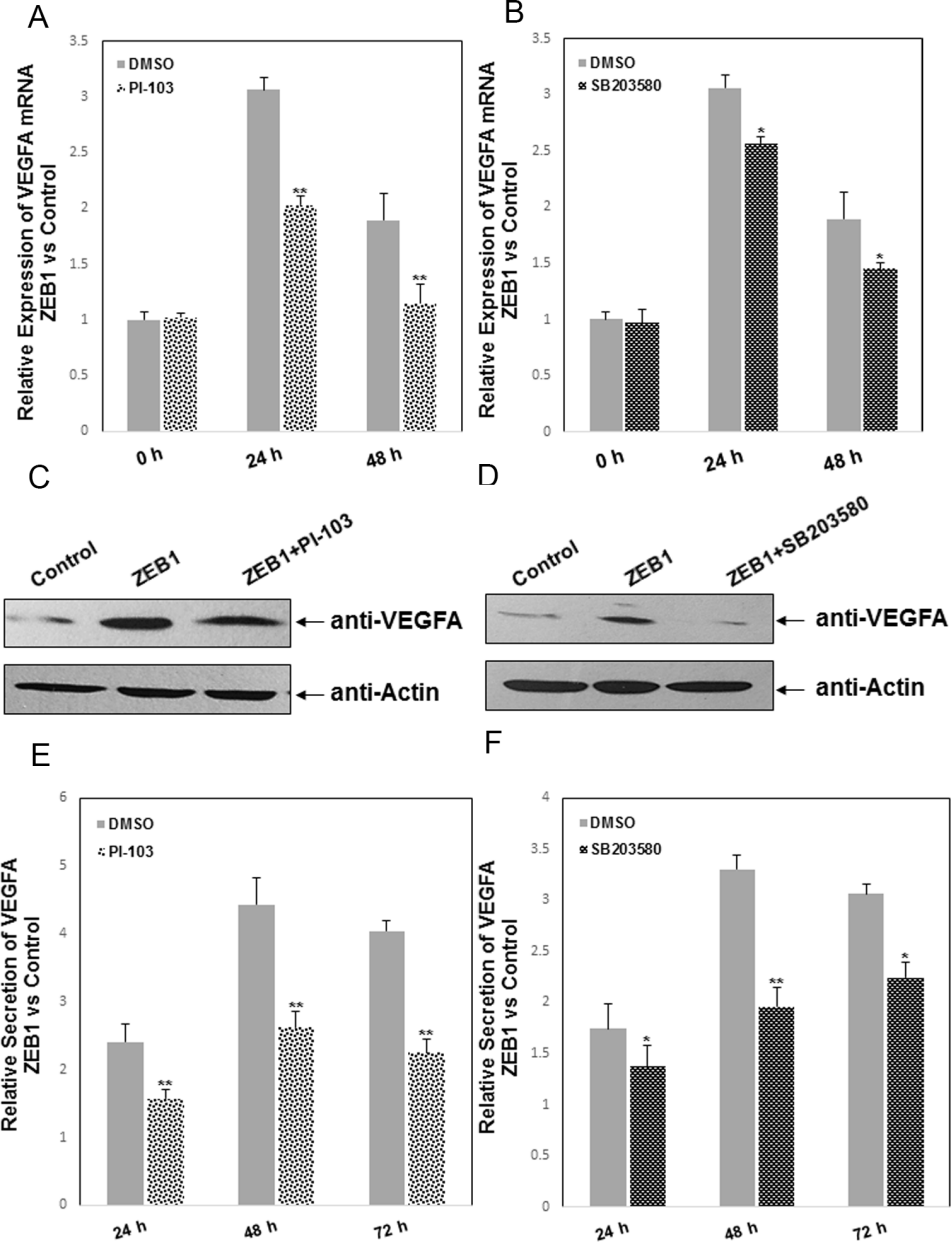
ZEB1 upregulates VEGFA expression by differentially regulating PI3K and p38 pathways. MDA-MB-231 cells were transiently transfected with the human ZEB1 expression plasmid or empty vector control, followed by treatment with PI-103 (10 μM) or SB203580 (10 μM). At the indicated time points, upregulation of VEGFA expression were verified by qPCR (A and B), Western blotting (C and D) and ELISA (E and F) in ZEB1-expressing versus control cells. GAPDH and actin were used to normalize VEGFA levels. *P < 0.05, **P < 0.01 vs respective control in Student’s *t*-test.

To further elucidate molecular changes, we proceeded to identify downstream signaling mechanisms for ZEB1-induced VEGFA expression. Considering that ZEB1 potentially functions through the NF-кB, PI3K and MAPKs pathways in breast cancer [32–34], MDA-MB-231 cells were transiently transfected with the ZEB1 expression plasmid or empty vector control, followed by treatment with BAY (NF-кB inhibitor), PI-103 (PI3K inhibitor), PD98059 (ERK inhibitor), SB203580 (p38 inhibitor), or SP600125 (JNK inhibitor). As shown in Fig 4A and 4B, quantitative PCR (qPCR) revealed that treatment with PI-103 or SB203580 significantly inhibited ZEB1-induced expression of VEGFA at the mRNA level in ZEB1-expressing versus control cells. However, inhibition of NF-кB, ERK and JNK signaling by BAY, PD98059 and SP600125 did not interfere with the VEGFA mRNA upregulation by ZEB1 (S2 Fig). Moreover, Western blotting and ELISA confirmed that ZEB1-induced upregulation of VEGFA protein expression was mediated via the PI3K and p38 pathways in MDA-MB-231 cells (Fig 4C to 4F).

### ZEB1 activates VEGFA transcription by increasing SP1 to its promoter

**Fig 5:**
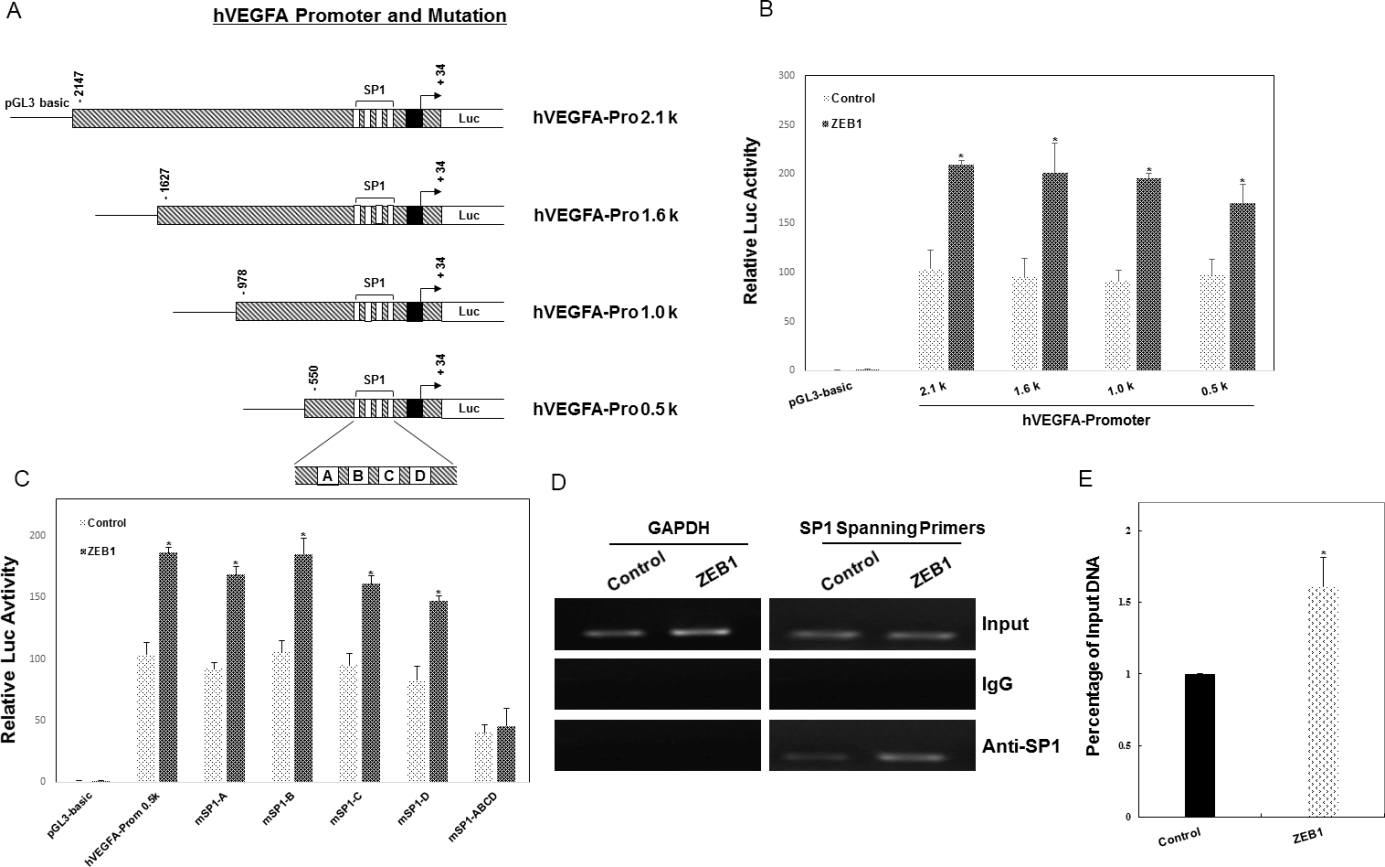
ZEB1 activates VEGFA transcription by recruiting SP1 to the endogenous VEGFA promoter. (A) Sequential deletion and mutation of SP1 elements on the human VEGFA promoter were fused to the luciferase reporter. (B) MDA-MB-231 cells were co-transfected with the ZEB1 expression plasmid (1 μg/well) and different wild-type VEGFA promoter luciferase reporter constructs (1 μg/well). Extract luciferase activities were determined 48 h after transfection using a Betascope analyzer. Luciferase values were normalized to Renilla activities. *P < 0.05 vs respective control in Student’s *t*-test. (C) MDA-MB-231 cells were co-transfected with ZEB1 expression plasmid and wild-type or mutant VEGFA promoter luciferase reporters. Extract luciferase activities were determined 48 h after transfection using a Betascope analyzer. Luciferase values are normalized with Renilla activities. *P < 0.05 vs respective control in Student’s *t*-test. (D) The association of SP1 with the proximal human VEGFA promoter was analyzed by ChIP assay, using polyclonal Ab against SP1 or unrelated IgG Ab. The amplified sequence of the VEGFA promoter fragment containing SP1 elements is shown. Input DNA amounts were confirmed by equal loading of chromatin. (E) Overexpression of ZEB1 significantly enhanced the recruitment of SP1 to the endogenous VEGFA promoter as confirmed by a quantitative ChIP assay. *P < 0.05 vs respective control in Student’s *t*-test.

We next assessed whether ZEB1 is a *bona fide* activator of VEGFA transcription using a reporter gene assay. As seen in Fig 5A and 5B, ZEB1 overexpression significantly increased the human VEGFA promoter activity of the wild-type -2147/+34 reporter by approximately 2-fold relative to the control. Furthermore, a series of truncated VEGFA promoter-reporter constructs were generated, shown in Fig 5A. The results showed that deletion of the -2147/-550 region of the VEGFA promoter does not affect its transactivation induced by ZEB1. We further performed a search using the transcription factor database TRANSFAC and identified 4 SP1 sites at positions -96/-88, -85/-77, -74/-66, and -60/-52 on the VEGFA promoter, which have been reported to mediate VEGF promoter activation in breast cancer [35]. SP1 sites were then manipulated by site-directed mutagenesis, individually or in combination (Fig 5A). The results of luciferase assays showed that site-directed mutagenesis of any individual SP1 site did not affect the ZEB1-induced activity of the VEGFA promoter. However, simultaneous mutations within all 4 SP1 sites (mSP1-ABCD) completely eliminated the stimulatory effect of ZEB1 on the promoter activity of VEGFA (Fig 5C). In addition, the quantitative ChIP assay indicated that ZEB1 overexpression resulted in a 1.6-fold increase in SP1 binding to the endogenous VEGFA promoter (Fig 5D and 5E), suggesting that overexpressed ZEB1 activates VEGFA transcription by recruitment of SP1 to the VEGFA promoter.

### Elevated expression of ZEB1 promotes angiogenesis *in vivo*

**Fig 6:**
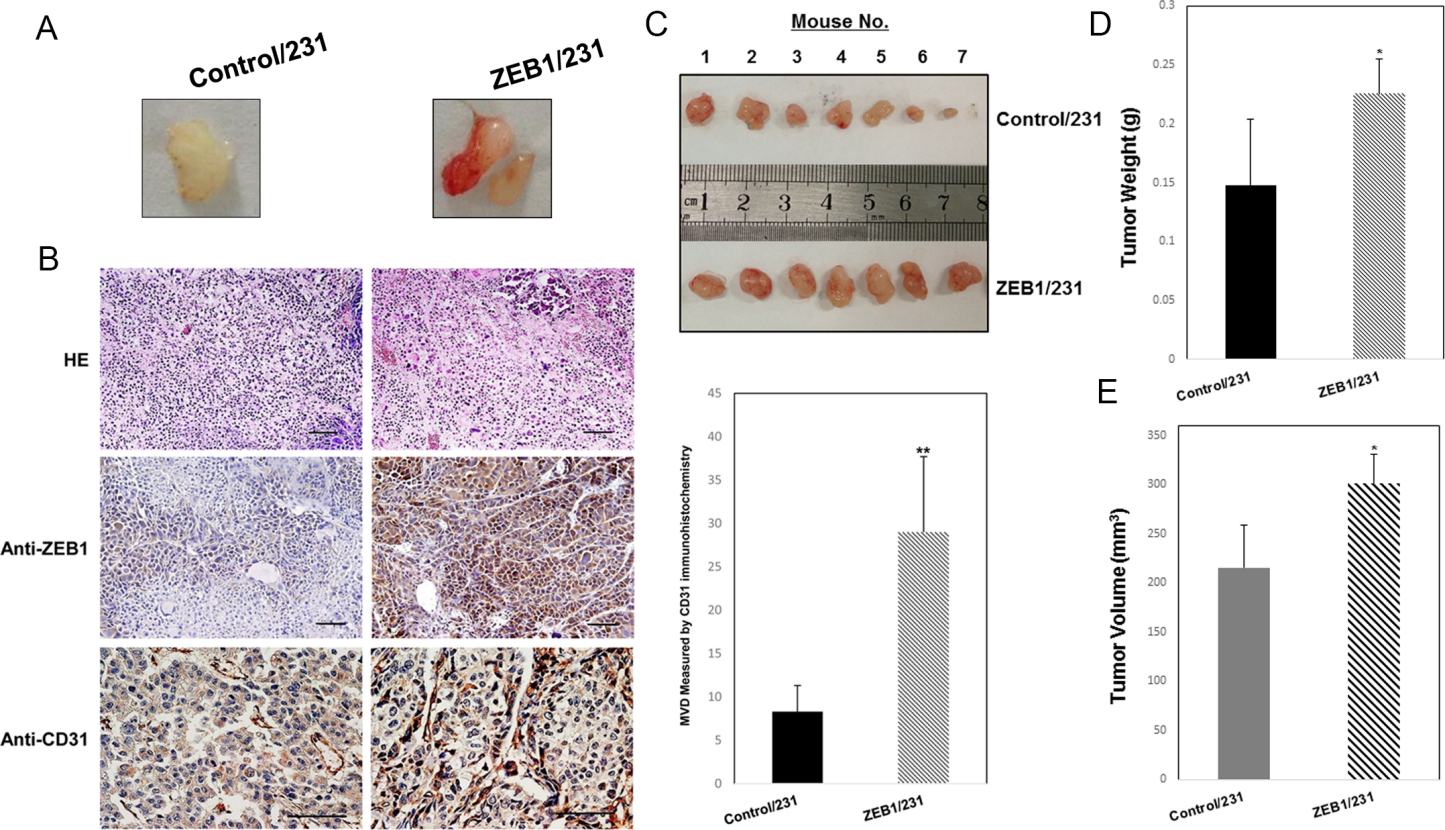
Elevated expression of ZEB1 promotes angiogenesis *in vivo* in a nude mouse xenograft model. (A) A mixture of Matrigel with ZEB1/231 or Control/231 cells was subcutaneously injected into the nude mice (n = 4). Resulting plugs were harvested 10 days later and photographed. (B) A total of 1.5 × 10^6^ ZEB1/231 or Control/231 cells were injected into the mammary fat pads of nude mice (n = 7). Tumor development was followed for 3 weeks, and then the mice were killed. Expression of ZEB1 and CD31 in breast cancer xenografts was examined by immunohistochemical staining. Scale bars, 25 μm. MVD was determined by CD31 staining. Five random fields in each tumor were counted for MVD. **P < 0.01 vs respective control in Student’s *t*-test. (C) Tumors from ZEB1/231 or Control/231 mice are shown and cross-sectional tumor diameters were measured. Approximate tumor weights (D) and volumes (E) were calculated as described in Materials and Methods. *P < 0.05 vs respective control in Student’s *t*-test.

Next, we determined whether elevated expression of ZEB1 in breast cancer cells influences tumor angiogenesis *in vivo*. We conducted Matrigel plug assays in nude mice using ZEB1/231 and Control/231 cells and harvested the plugs 10 days after implantation. Macroscopic examination revealed that Matrigel plugs containing ZEB1/231 cells exhibited more blood vessels than plugs with Control/231 (Fig 6A). Moreover, ZEB1/231 or Control/231 cells were injected into the mammary fat pads of female nude mice to establish a nude mouse xenograft model. Immunohistochemical staining confirmed the upregulation of ZEB1 expression in tumors from ZEB1/231 mice (Fig 6B). Our results were further supported by CD31 immunostaining of those tumors, which showed a significantly increased microvascular density (MVD) in tumors from ZEB1/231 mice compared with those from Control/231 mice (Fig 6B). Additionally, MDA-MB-231 cells stably overexpressing ZEB1 formed larger tumors which grew faster than the control-transfected cells (Fig 6C). Tumor volumes and weights in mice injected with ZEB1/231 cells were significantly higher than in those injected with Control/231 cells (Fig 6D and 6E), consistent with our previous study that ZEB1 promotes tumorigenesis of breast cancer [34,36]. Collectively, these data demonstrate that ZEB1 has the capacity to induce the formation of new blood vessels *in vivo*, and thus facilitates tumorigenesis in breast cancer.

## Discussion

Angiogenesis is a critical event, vital to the growth of tumors and VEGF is a key factor in tumor angiogenesis. Therefore, understanding the regulatory mechanisms of VEGF expression in cancer cells may have important implications for novel therapies to combat various cancers. In this study, we showed that ectopic expression of ZEB1 resulted in significant upregulation of VEGFA synthesis in MDA-MB-231 breast cancer cells, thus promoting tumor angiogenesis *in vitro* and *in vivo*. We have confirmed that tumors with high levels of ZEB1 exhibit a dramatically increased blood vessel density in breast cancer specimens. On the basis of these results, we propose a model in which elevated expression of ZEB1 in breast cancer cells activates the secretion of VEGFA protein and creates a chemotactic gradient to promote angiogenic stimulation.

We previously reported that ZEB1 promotes breast cancer migration and invasion by inducing EMT [36]. In the current study, we have additionally identified an important role for ZEB1 in tumor progression; ie, the stimulation of angiogenesis. We found that the mechanism underlying ZEB1-stimulated angiogenesis is the induction of VEGFA production by breast cancer cells. This regulation of VEGFA by ZEB1 was demonstrated not only in cells representing basal breast cancer cells (eg, MDA-MB-231; estrogen-receptor negative) but also in cells representing luminal breast cancer cells (eg, MCF-7; estrogen-receptor positive; S3 Fig), suggesting that ZEB1-induced angiogenesis may be a general mechanism in this disease. The pro-angiogenic activity of ZEB1 is consistent with previous reports that an increased ZEB1 level is observed in advanced human breast cancer tissue [37,38]. This is also consistent with our observation here that increased tumor development and growth are associated with elevated expression of ZEB1 in animal models of breast cancer.

We found that ZEB1 enhances tumor angiogenesis *in vivo* both in a Matrigel plug assay and in a nude mouse xenograft model. Several possible mechanisms may underlie these findings. First, as presented in this study, ZEB1 may induce VEGFA production by breast cancer cells through activation of the PI3K and p38 pathways. Mechanically, Yang *et al*. reported that in lung adenocarcinoma cells from *Kras/Tp53*-mutant animals and human lung cancer cell lines, ZEB1 activates PI3K by depressing miR-200 targets, including amphiregulin (*AREG*), betacellulin (*BTC*), and *GATA6* [33]. Our previous study showed that ectopic expression of ZEB1 induces breast cancer metastasis through activation of the MAPK signaling, including increased phosphorylation of p38 [34]. Possibly, ZEB1 functions to inhibit PP2A (protein phosphatase 2A), which consequently promotes p38 phosphorylation in breast cancer cells [39,40]. Second, both ZEB1 and VEGFA may induce the production of matrix metalloproteinases (MMPs), such as MMP-2 and MMP-9, in breast cancer cells [34,41]. In this case, the more active pool of MMP-2 and MMP-9 may be available to facilitate angiogenesis by remodeling extracellular matrix as well as by releasing matrix-bound angiogenic factors [42,43]. Considering that HIF-1 is a critical transcription factor for the regulation of VEGF [44,45] and induces ZEB1 expression under both normoxia and hypoxia conditions during tumorigenesis [46–48], a third possibility is that ZEB1 may act in a feedback-loop with HIF-1. Joseph *et al*. reported that hypoxia-induced activation of HIF-1α-ZEB1 signaling axis contributes to the mesenchymal shift and invasion in glioblastoma cells [46]. Similarly, in breast cancer and renal clear cell carcinoma under normoxia condition, HIF-1 increases ZEB1 expression and thus regulates EMT [47,48]. On the other hand, the regulatory role of ZEB1 on HIF-1 seems controversial. Clarhaut *et al*. found that ectopic ZEB1 inhibits SEMA3F and leads to an increase in HIF-1 protein in lung cancer [49]. However, ZEB1 knockdown in glioblastoma cells shows no effect on HIF-1 expression [46]. This is consistent with our present study that compared with the empty vector control, overexpression of ZEB1 has no effect on HIF-1 activity in MDA-MB-231 cells (data not shown), indicating that HIF-1 may act upstream of ZEB1 in breast cancer angiogenesis.

High levels of VEGF expression in many cell lines under normoxic conditions implicate additional factors regulating VEGF [50]. For example, activation of both HER-2/Neu and PI3K/Akt has been shown to increase VEGF transcription. This effect was mediated by transcriptional activation of the VEGF promoter via SP1 binding sites [51]. A recent report also demonstrated that Twist, which is a member of the basic helix-loop-helix transcription factor family, contributes to the positive regulation of VEGF in metastatic breast cancer. Twist-mediated angiogenesis in clinical samples correlates with higher expression of VEGF [52]. However, the underlying cellular mechanism concerning Twist-induced activation of VEGF remains to be elucidated. In this report, we have provided further evidence of a novel regulatory mechanism demonstrating that ZEB1 mediates the stimulation of VEGF synthesis and which may contribute to tumor growth and angiogenesis in breast cancer. Using luciferase and ChIP assays, we revealed that overexpression of ZEB1 in MDA-MB-231 cells results in increased recruitment of SP1 to the VEGF promoter, where it binds and activates transcription. A recent study reported that SP1 is a direct target of miR-200b and is involved in miR-200b-induced suppression of breast cancer cell growth [53]. Considering that a double-negative feedback loop between ZEB1 and the miRNA-200 family has been demonstrated in tumorigenesis [54,55], it is likely that elevated ZEB1 inhibits miR-200b and thus promotes SP1 levels to recruit to the VEGF promoter. On the other hand, activation of the PI3K and p38 signaling have been implicated in the induction of SP1 activity during tumorigenesis and angiogenesis [51,56,57]. For example, SP1 shows increased phosphorylation under conditions of PI3K/Akt activation, resulting in transcriptional activation of VEGF expression in glioblastoma and prostate carcinoma cells [51]. These observations support our notion that ZEB1 induces VEGF production by breast cancer cells through activation of the PI3K and p38 pathways, which effect is mediated via increased activity and recruitment of SP1 to the VEGF promoter.

In summary, these findings mechanistically link ZEB1 with increased angiogenesis in breast cancer and identify a novel mechanism by which ZEB1 may promote breast cancer progression. Taken together with its EMT-inducing activity, these data raise the possibility that ZEB1 may be a potential therapeutic target for this disease.

## Materials and Methods

### Plasmid construction

Generation of the full-length human ZEB1 expression plasmid has previously been described [36]. Human VEGFA (-2147/+34) promoters were amplified by PCR from the genomic DNA of human blood cells and cloned into the pGL3-basic vector (Promega). The mutagenesis of SP1 elements on the VEGFA promoter was performed using the Quick Change Site-Directed Mutagenesis Kit (Stratagene).

### Preparation of short hairpin RNAs (shRNAs)

The shRNA target sequence for ZEB1 was 5’-CGGCGCAATAACGTTACAAAT-3’. Sense and antisense oligonucleotides with internal loops were synthesized, annealed and ligated into pLKO.1 (Sigma-Aldrich) to construct the ZEB1-specific shRNA expression plasmid (shZEB1). pLKO.1 expressing scrambled shRNAs (shControl) were used as controls.

### Cell culture and transfection

MDA-MB-231 and MCF-7 human breast cancer cells were maintained in high glucose Dulbecco’s Modified Eagle Medium (DMEM) supplemented with 10% fetal bovine serum (FBS), penicillin, and streptomycin (Invitrogen). HUVECs were maintained in RPMI 1640 supplemented with 10% FBS, penicillin, and streptomycin. Cells were transfected using TurboFect Transfection Reagent (Roche) according to the manufacturer’s protocols.

The human ZEB1 expression plasmid (G418 resistance) or shRNA plasmid targeting ZEB1 (puromycin resistance) was introduced into MDA-MB-231 or MCF-7 cells by transient transfection. Drug-resistant clones were isolated over a period of 3–4 weeks. Overexpression or knockdown of ZEB1 was confirmed by Western blotting.

### Western blotting and antibodies

Preparation of total cell extracts and Western blotting with appropriate antibodies was performed as previously described [36]. The following antibodies were used: goat polyclonal Ab against ZEB1 (ab81972; Abcam) at dilution of 1:1000, rabbit polyclonal Ab against VEGFA (19003-1-AP; Proteintech) at dilution of 1:1000, rabbit polyclonal Ab against VEGFC (22601-1-AP; Proteintech) at dilution of 1:500, and mouse monoclonal Ab against actin (A-4700; Sigma) at dilution of 1:1000.

### Collection of conditioned medium

Stable MDA-MB-231 and MCF-7 transfectants (1.5 × 10^6^ cells) were seeded into T-75 flasks and cultured in DMEM with 0.5% FBS. Conditioned medium was collected after 48–72 h, normalized by the content of genomic DNA by adding DMEM, as described by Dai *et al*. [58], and concentrated by approximately 80~100 folds using Centrifugal Filter Units (Millipore, UFC801024).

### Tube formation assay

A pre-chilled 24-well plate was coated with 200 μL of 10 mg/mL Matrigel (Sigma-Aldrich) and allowed to solidify at 37°C for 1 h. HUVECs (5 × 10^3^ cells/well) were seeded on the Matrigel and cultured in the absence or presence of human anti-VEGFA- or anti-VEGFC-neutralized Ab (1 μg/mL) along with conditioned medium derived from stable MDA-MB-231 and MCF-7 transfectants. After incubation at 37°C for 16 h, 5 randomly chosen fields were counted and photographed (Olympus).

### RNA extraction and quantitative RT-PCR

Using TRIzol Reagent (Invitrogen), total RNA was extracted from MDA-MB-231 and MCF- cells that were transiently transfected with the human ZEB1 expression plasmid or empty vector. Total RNA (0.5 μg) from each sample was used for first-strand cDNA synthesis using M-MLV Reverse Transcriptase (Promega). The specific products of human VEGFA were amplified by qPCR and verified using EvaGreen (Biotium). GAPDH was used as an internal control.

### RT^2^ Profiler PCR array

Total RNA was extracted from MDA-MB-231 cells stably transfected with the human ZEB1 expression plasmid or empty vector. For PCR array experiments, an RT^2^ Profiler custom PCR array was used to simultaneously examine the mRNA levels of 84 genes closely associated with angiogenesis, including 5 housekeeping genes, in 96-well plates following the manufacturer’s protocol (PAHS-049Z, QIAGEN). Briefly, first-strand cDNAs were synthesized from 1 μg of total RNA using the TaqMan RT reagent kit (QIAGEN) according to the manufacturer’s protocol. The reaction mixtures (25 μL) were incubated at 25°C for 10 min, followed by incubation at 48°C for 30 min and 95°C for 5 min, then cooled on ice. Arrays were performed independently at least 3 times for each cell line; values were obtained for the threshold cycle (Ct) for each gene and normalized using the average of 5 housekeeping genes on the same array (ACTB, B2M, GAPDH, HPRT1, and RPLP0). The Ct values for housekeeping genes and a dilution series of ACTB were monitored for consistency between the arrays. The change (ΔCt) between ZEB1/231 and Control/231 was determined using the formula, ΔCt = Ct(ZEB1/231)—Ct(Control/231). The fold-change was determined using the formula, fold-change = 2^(-ΔCt)^. The resulting values were reported as fold-change; only genes showing two-fold or greater change were considered. Negative controls ensured absence of DNA contamination and defined thresholds for determining absence versus presence of expression.

### Estimation of VEGFs protein by ELISA

Amounts of VEGFA or VEGFC protein in the extracellular medium were determined using protocols provided by the ELISA kit manufacturer (R&D Systems). Following adsorption of the goat anti-human VEGFA Ab or the goat anti-human VEGFC Ab, plates was washed and incubated with a fixed amount of total protein from the medium. Plates were then incubated with biotinylated anti-goat Ab, washed, and further incubated with streptavidin-horseradish peroxidase. The chromogen tetramethylbenzidine was added, and absorbance at 450 nm was determined using a microplate reader.

### Tissue samples

Fresh breast cancer tissue of invasive ductal carcinomas were obtained from Tianjin Medical University Cancer Institute and Hospital. Tissue samples were collected from a total of 228 subjects. All patients were diagnosed with histologically confirmed breast cancer and were recruited from the same department. The tissue specimen collection was approved by the Internal Review and Ethics Boards at the Medical College of Nankai University, and the written informed consent was obtained from each patient.

### Immunohistochemical analysis and assessment of MVD

Immunohistochemical analysis of paraffin-embedded sections was performed using the Envision Kit (Dako) following the manufacturer’s protocols. Sections were boiled in retrieval solutions to expose antigens. We applied rabbit polyclonal Ab against ZEB1 (ab87280; Abcam), rabbit polyclonal Ab against VEGFA (19003-1-AP; Proteintech), rabbit polyclonal Ab against CD31 (11265-1-AP; Proteintech), and control primary Abs to the sections at 1:100 dilution. Slides were counterstained with hematoxylin, dehydrated, and mounted. Immunostaining was independently evaluated by 2 pathologists.

To calculate MVD, the 5 most-vascularized areas of the tumor were selected and mean values obtained by counting vessels. A single microvessel was defined as a discrete cluster of cells positive for CD31 staining, with no requirement for the presence of a lumen.

### Luciferase assay

MDA-MB-231 cells were co-transfected with the wild-type or mutant human VEGFA promoters and ZEB1 expression plasmid in 24-well plates. Lysates were prepared at 48 h after transfection, and luciferase activities were measured using the Dual-Luciferase Reporter Assay System (Promega) according to the manufacturer’s protocols. The luciferase activities were normalized to the values for Renilla luciferase.

### Chromatin immunoprecipitation

Chromatin immunoprecipitation (ChIP) assays were performed using reagents commercially obtained from Upstate, essentially according to the manufacturer’s instructions. The Abs used in these experiments were rabbit monoclonal Ab against SP1 (#9389S; CST) and anti-rabbit normal IgG (sc-2345, Santa Cruz). The fragment of human VEGFA promoter containing the SP1 elements in immunoprecipitates was determined by qPCR.

### Animals

All experimental procedures involving animals were performed according to the institutional ethical guidelines for animal experiments and approved by the Ethics Committee for Animal Use at the Medical College of Nankai University. Euthanasia was performed by introduction of 100% carbon dioxide into a bedding-free cage initially containing room air with the lid closed at a rate sufficient to induce rapid anesthesia, with death occurring within 2.5 minutes. Using a calibrated anesthetic delivery machine, mice were induced into anesthesia at a dose of 4% isoflurane, then maintained at a surgical plane by continuous inhalation of 2% isoflurane. All efforts were made to ameliorate animal suffering and to reduce the number of animals used.

### Matrigel plug assay

BALB/c nude mice (mean body weight: 22.8 ± 0.5 g; n = 4) were given subcutaneous injections of 200 mL of a 3:1 mixture of growth factor-reduced Matrigel (BD Biosciences) and 2 × 10^6^ cells in DMEM. After 10 days, plugs were harvested, imaged, and snap frozen in liquid nitrogen in the presence of optimum cutting temperature (OCT) compound (Tissue-Tek) before sectioning. Frozen Matrigel sections were fixed in cold acetone and immunostained with phycoerythrin-conjugated anti-CD31 Ab for MVD measurement.

### Tumor xenograft experiments

Stable MDA-MB-231 transfectants were collected and suspended in 50 μL of PBS at a concentration of 3 × 10^7^ cells/mL, then injected into the mammary fat pads of female BALB/c nude mice (mean body weight: 23.6 ± 0.6 g; n = 7). The mice were killed when tumor masses were detected 3 weeks after surgery. The tumor volume (*V*) was calculated by measuring the length (*L*) and width (*W*) of the tumor with calipers and using the formula *V* = (*L* × *W*^*2*^) × 0.5. Tumor tissues were also processed and sectioned for histological evaluation.

### Statistical analysis

SPSS 17.0 software (SPSS) was used for statistical analysis. The data from all the experiments are presented as means ± SD and represent three independent experiments. One-way analysis of variance (ANOVA) was used to compare means between treatment groups and Tukey’s HSD (honestly significant difference) test was used to evaluate the statistically significant differences between groups. Where appropriate, Student’s *t*-test for unpaired observations was applied. A *p*-value < 0.05 was considered significant.

## Supporting Information

S1 Fig ZEB1 induces tumor angiogenesis of breast cancer by altering the levels of angiogenic regulators. MDA-MB-231 cells were stably transfected with the human ZEB1 expression plasmid (ZEB1/231) or empty vector control (Control/231). Expression of EFNB2 (A), VEGFA (B) VEGFC (C), PDGFA (D), and IL6 (E) were examined by qPCR and Western blotting. GAPDH and actin were used to normalize the individual levels. *P < 0.05 vs. respective control in Student’s *t*-test.
(DOCX)

S2 Fig ZEB1 upregulates VEGFA expression by differentially regulating PI3K and p38 pathways. MDA-MB-231 cells were transiently transfected with the human ZEB1 expression plasmid or empty vector control, followed by treatment with BAY (A), PD98059 (B) or SP600125 (C). At the indicated time points, upregulation of VEGFA mRNA were verified by qPCR. GAPDH was used to normalize VEGFA levels.
(DOCX)

S3 Fig Ectopic expression of ZEB1 promotes MCF-7-mediated angiogenesis *in vitro*. (A) MCF-7 cells were transiently transfected with the human ZEB1 expression plasmid or empty vector control. At the indicated time points, expression of ZEB1 protein was verified by Western blotting. Actin was used to normalize ZEB1 levels. Upregulation of VEGFA mRNA and protein were verified by qPCR (B), Western blotting (C) and ELISA (D) at the indicated time points. GAPDH and actin were used to normalize the VEGFA levels. *P < 0.05, **P < 0.01 vs. respective control in one-way ANOVA followed by Tukey’s HSD test. (E) MCF-7 cells were stably transfected with the human ZEB1 expression plasmid (ZEB1/MCF-7) or empty vector control (Control/MCF-7). HUVECs cultured in ZEB1/MCF-7- or Control/MCF-7-derived conditioned medium were subjected to a tube formation assay and photographed. (F) Quantification of tube formation was expressed in length of capillary tubes formed per mm^2^. **P < 0.01 vs. respective control in Student’s *t*-test.
(DOCX)

S1 File Supplementary materials and methods. (DOCX)
