# Supplementary material for: ZEB1 Upregulates VEGF Expression and Stimulates Angiogenesis in Breast Cancer
Source: PLoS One. 2016 Feb 16;11(2):e0148774. doi: 10.1371/journal.pone.0148774 (PMC4755590; doi:10.1371/journal.pone.0148774)
Supplement: S1 Fig — MDA-MB-231 cells were stably transfected with the human ZEB1 expression plasmid (ZEB1/231) or empty vector control (Control/231). Expression of EFNB2 (A), VEGFA (B) VEGFC (C), PDGFA (D), and IL6 (E) were examined by qPCR and Western blotting. GAPDH and actin were used to normalize the individual levels. *P < 0.05 vs. respective control in Student’s t-test. (DOCX) [file pone.0148774.s001.docx]

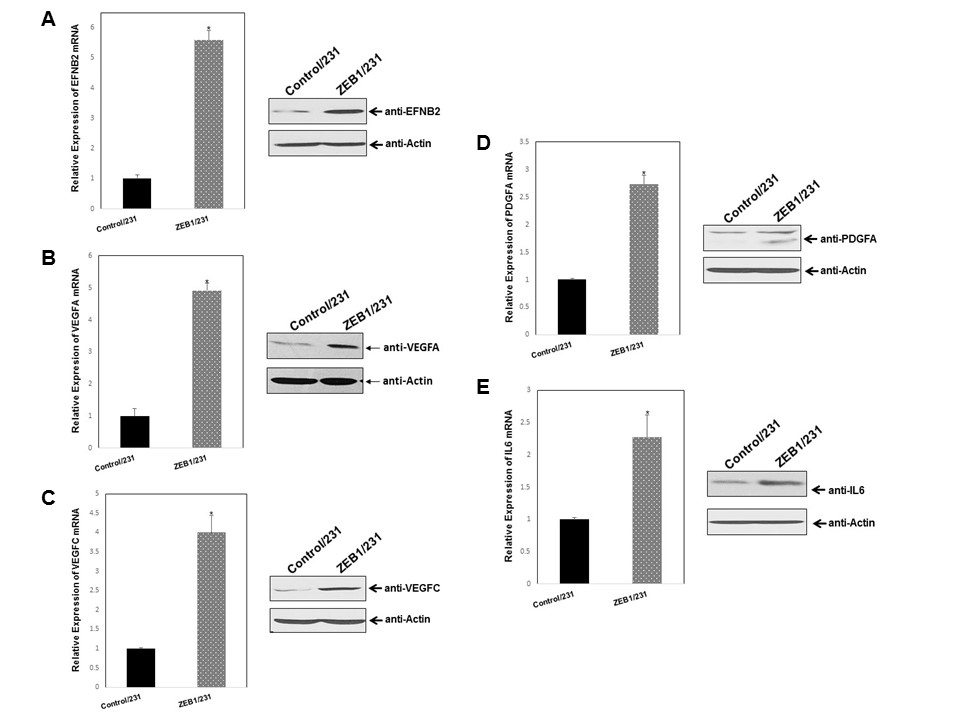


**S1 Fig. ZEB1 induces tumor angiogenesis of breast cancer by altering the levels of angiogenic regulators.** MDA-MB-231 cells were stably transfected with the human ZEB1 expression plasmid (ZEB1/231) or empty vector control (Control/231). Expression of EFNB2 (A), VEGFA (B) VEGFC (C), PDGFA (D), and IL6 (E) were examined by qPCR and Western blotting. GAPDH and actin were used to normalize the individual levels. **P* < 0.05 vs respective control in Student’s *t*-test.
