# Supplementary material for: ZEB1 Upregulates VEGF Expression and Stimulates Angiogenesis in Breast Cancer
Source: PLoS One. 2016 Feb 16;11(2):e0148774. doi: 10.1371/journal.pone.0148774 (PMC4755590; doi:10.1371/journal.pone.0148774)
Supplement: S2 Fig — MDA-MB-231 cells were transiently transfected with the human ZEB1 expression plasmid or empty vector control, followed by treatment with BAY (A), PD98059 (B) or SP600125 (C). At the indicated time points, upregulation of VEGFA mRNA were verified by qPCR. GAPDH was used to normalize VEGFA levels. (DOCX) [file pone.0148774.s002.docx]

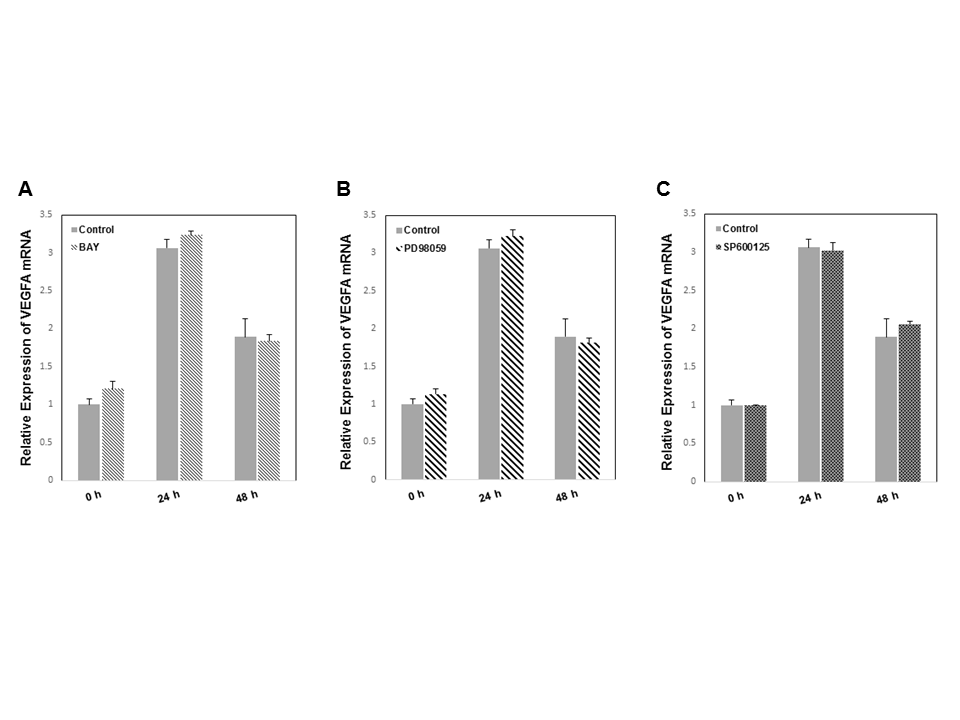


**S2 Fig. ZEB1 upregulates VEGFA expression by differentially regulating PI3K and p38 pathways.** MDA-MB-231 cells were transiently transfected with the human ZEB1 expression plasmid or empty vector control, followed by treatment with BAY (A), PD98059 (B) or SP600125 (C). At the indicated time points, upregulation of VEGFA mRNA were verified by qPCR. GAPDH was used to normalize VEGFA levels.
