# Supplementary material for: ZEB1 Upregulates VEGF Expression and Stimulates Angiogenesis in Breast Cancer
Source: PLoS One. 2016 Feb 16;11(2):e0148774. doi: 10.1371/journal.pone.0148774 (PMC4755590; doi:10.1371/journal.pone.0148774)
Supplement: S3 Fig — (A) MCF-7 cells were transiently transfected with the human ZEB1 expression plasmid or empty vector control. At the indicated time points, expression of ZEB1 protein was verified by Western blotting. Actin was used to normalize ZEB1 levels. Upregulation of VEGFA mRNA and protein were verified by qPCR (B), Western blotting (C) and ELISA (D) at the indicated time points. GAPDH and actin were used to normalize the VEGFA levels. *P < 0.05, **P < 0.01 vs. respective control in one-way ANOVA followed by Tukey’s HSD test. (E) MCF-7 cells were stably transfected with the human ZEB1 expression plasmid (ZEB1/MCF-7) or empty vector control (Control/MCF-7). HUVECs cultured in ZEB1/MCF-7- or Control/MCF-7-derived conditioned medium were subjected to a tube formation assay and photographed. (F) Quantification of tube formation was expressed in length of capillary tubes formed per mm2. **P < 0.01 vs. respective control in Student’s t-test. (DOCX) [file pone.0148774.s003.docx]

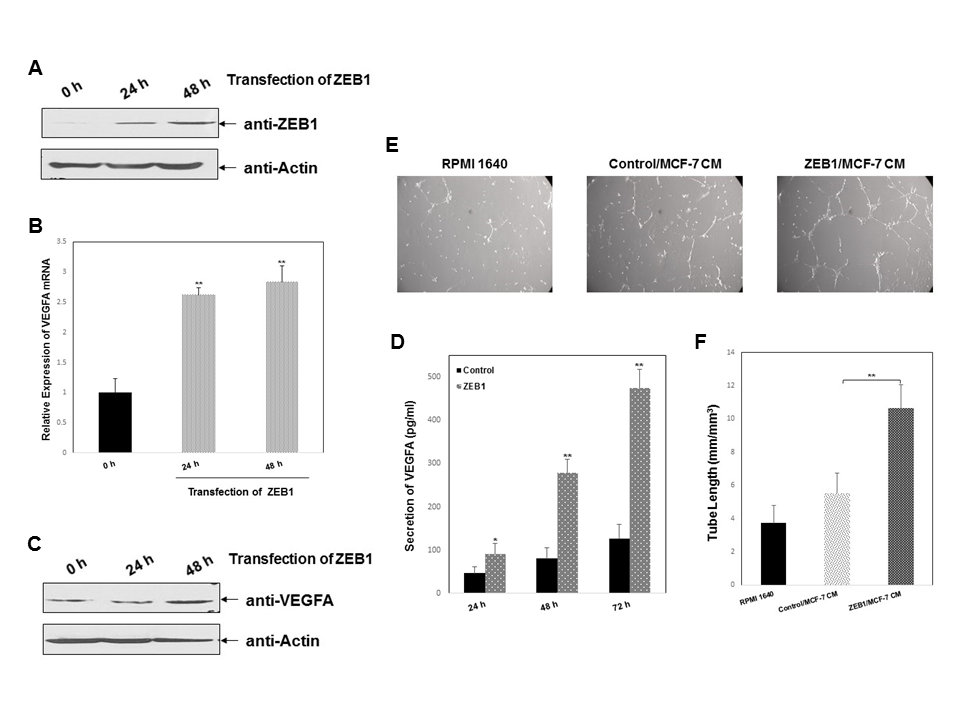


**S3 Fig. Ectopic expression of ZEB1 promotes MCF-7-mediated angiogenesis *in vitro.*** (A) MCF-7 cells were transiently transfected with the human ZEB1 expression plasmid or empty vector control. At the indicated time points, expression of ZEB1 protein was verified by Western blotting. Actin was used to normalize ZEB1 levels. Upregulation of VEGFA mRNA and protein were verified by qPCR (B), Western blotting (C) and ELISA (D) at the indicated time points. GAPDH and actin were used to normalize VEGFA levels. **P* < 0.05, ***P* < 0.01 vs respective control in one-way ANOVA followed by Tukey’s HSD test. (E) MCF-7 cells were stably transfected with the human ZEB1 expression plasmid (ZEB1/MCF-7) or empty vector control (Control/MCF-7). HUVECs cultured in ZEB1/MCF-7- or Control/MCF-7-derived conditioned medium were subjected to a tube formation assay and photographed. (F) Quantification of tube formation was expressed in length of capillary tubes formed per mm^2^. ***P* < 0.01 vs respective control in Student’s *t*-test.
