# Supplementary material for: ZEB1 Upregulates VEGF Expression and Stimulates Angiogenesis in Breast Cancer
Source: PLoS One. 2016 Feb 16;11(2):e0148774. doi: 10.1371/journal.pone.0148774 (PMC4755590; doi:10.1371/journal.pone.0148774)
Supplement: S1 File — (DOCX) [file pone.0148774.s004.docx]

**Supplementary materials and methods**

**PCR primers**

| VEGFA Promoter Construction | |
| --- | --- |
| wt 2.1 k forward | 5’-ATAGGTACCGTTGGCTCTTTTAGGGGCTG-3’ |
| wt 2.1 k reverse | 5’-AGTAAGCTTCCACGACCTCCGAGCTACCC-3’ |
| mSP1-A forward | 5’-CGCCTGTCCCCGAACCCCGGGGCGGG-3’ |
| mSP1-A reverse | 5’-CCCGCCCCGGGGTTCGGGGACAGGCG-3’ |
| mSP1-B forward | 5’-CCGCCCCCCGGTTCGGGCCGGGGG-3’ |
| mSP1-B reverse | 5’-CCCCCGGCCCGAACCGGGGGGCGG-3’ |
| mSP1-C forward | 5’-GGGCGGGCCGGGTTCGGGGTCCCGGC-3’ |
| mSP1-C reverse | 5’-GCCGGGACCCCGAACCCGGCCCGCCC-3’ |
| mSP1-D forward | 5’-GGTCCCGGCGGTTCGGAGCCATGC-3’ |
| mSP1-D reverse | 5’-GCATGGCTCCGAACCGCCGGGACC-3’ |
| Quantitative RT-PCR | |
| human VEGFA forward | 5’-GCTGTTATCTGGGGCGAGGG-3’ |
| human VEGFA reverse | 5’-GGTGGGACCATGAGTGCTGC-3’ |
| human EFNB2 forward | 5’-TATGCAGAACTGCGATTTCCAA-3’ |
| human EFNB2 reverse | 5’-TGGGTATAGTACCAGTCCTTGTC-3’ |
| human VEGFC forward | 5’-GAGGAGCAGTTACGGTCTGTG-3’ |
| human VEGFC reverse | 5’-TCCTTTCCTTAGCTGACACTTGT-3’ |
| human PDGFA forward | 5’-GCAAGACCAGGACGGTCATTT-3’ |
| human PDGFA reverse | 5’-GGCACTTGACACTGCTCGT-3’ |
| human IL6 forward | 5’-ACTCACCTCTTCAGAACGAATTG-3’ |
| human IL6 reverse | 5’-CCATCTTTGGAAGGTTCAGGTTG-3’ |
| Quantitative CHIP | |
| Forward | 5’-GGCTGTGAACCTTGGTGGGG-3’ |
| Reverse | 5’-CCACGACCTCCGAGCTACCC-3’ |

**Western blotting and Abs**

Total cell extract preparation and western blotting with the appropriate antibodies were performed as previously described [36]. The following Abs were used: rabbit monoclonal Ab against EFNB2 (ab150411; Abcam) at dilution of 1:1000, rabbit polyclonal Ab against VEGFA (19003-1-AP; Proteintech) at dilution of 1:1000, rabbit polyclonal Ab against VEGFC (22601-1-AP; Proteintech) at dilution of 1:500, rabbit polyclonal Ab against PDGFA (22011-1-AP; Proteintech) at dilution of 1:800, rabbit polyclonal Ab against IL6 (ab154367; Abcam) at dilution of , and mouse monoclonal Ab against actin (A-4700; Sigma) at 1:1000 dilution.
